# Naturally acquired immune responses to malaria vaccine candidate antigens MSP3 and GLURP in Guahibo and Piaroa indigenous communities of the Venezuelan Amazon

**DOI:** 10.1186/1475-2875-11-46

**Published:** 2012-02-15

**Authors:** Andreas Baumann, Magda M Magris, Marie-Luz Urbaez, Sarai Vivas-Martinez, Rommy Durán, Tahidid Nieves, Meral Esen, Benjamin G Mordmüller, Michael Theisen, Luisana Avilan, Wolfram G Metzger

**Affiliations:** 1 Servicio Autónomo Centro Amazónico para la Investigación y Control de Enfermedades Tropicales ‚Simón Bolívar'(SACAICET), Puerto Ayacucho, Estado Amazonas, Venezuela; 2 Cátedra de Salud Pública. Escuela de Medicina Luis Razetti, Universidad Central de Venezuela, Caracas, Venezuela; 3 Department of Clinical Biochemistry and Immunology, Statens Serum Institute and Centre for Medical Parasitology at the Department of International Health, Immunology and Microbiology, University of Copenhagen, Copenhagen, Denmark; 4 Departamento de Biologia, Facultad de Ciencias, Universidad de Los Andes, Merida, Venezuela; 5 Institut für Tropenmedizin, Eberhard Karls Universität Tübingen, Tübingen, Germany

## Abstract

**Background:**

Malaria transmission in most of Latin America can be considered as controlled. In such a scenario, parameters of baseline immunity to malaria antigens are of specific interest with respect to future malaria eradication efforts.

**Methods:**

A cross-sectional study was carried out in two indigenous population groups in Amazonas/Venezuela. Data from the regional malaria documentation system were extracted and participants from the ethnic groups of the Guahibo (n = 180) and Piaroa (n = 295) were investigated for the presence of *Plasmodium *parasites and naturally acquired antibodies to *Plasmodium falciparum *antigens in serum. The GMZ2 vaccine candidate proteins MSP3 and GLURP were chosen as serological markers.

**Results:**

The incidence of *P. falcip*arum in both communities was found to be less than 2%, and none of the participants harboured *P. falciparum *at the time of the cross-sectional. Nearly a quarter of the participants (111/475; 23,4%) had positive antibody titres to at least one of the antigens. 53/475 participants (11.2%) were positive for MSP3, and 93/475 participants (19.6%) were positive for GLURP. High positive responses were detected in 36/475 participants (7.6%) and 61/475 participants (12.8%) for MSP3 and GLURP, respectively. Guahibo participants had significantly higher antibody titres than Piaroa participants.

**Conclusions:**

Considering the low incidence of *P. falciparum*, submicroscopical infections may explain the comparatively high anti-*P. falciparum *antibody concentrations.

## Background

To date, malaria is among the top ten causes of death in low-income countries [1]. In Venezuela, particularly the southern states of Bolivar and Amazonas are affected by malaria transmission. However, mortality is insignificant and malaria can be considered as controlled [2]. Amazonas (180,145 km²) covers an area nearly twice as big as Portugal and is inhabited by approximately 150,000 persons. Roughly half of the population is of indigenous origin and - although the absolute number of cases is low - Amazonas has the highest malaria incidence per capita in Venezuela (2007: 68.4 cases/1,000 inhabitants). Overall, the main malaria species is *Plasmodium vivax *(ca. 80%) followed by *Plasmodium falciparum *(*ca *20%, with declining tendency). *Plasmodium malariae *is only occasionally detected [3,4].

As drug resistance continues to be a major problem, an effective malaria vaccine against *P. falciparum *would be a powerful tool in the control of malaria [5,6]. The vaccine candidate GMZ2 is a fusion protein of *P. falciparum *merozoite surface protein 3 (MSP3) and glutamate rich protein (GLURP), which has been evaluated during phase I trials as a promising vaccine candidate in Germany and Africa [7,8]. Antibodies against both antigens have been shown to provide partial protection in *Saimiri sciureus *monkeys [9] and were associated with protection from human clinical malaria [10]. However, no data are available in respect to naturally acquired immunity to MSP3 and GLURP from populations of Latin America [11]. This is of interest since a malaria vaccine such as GMZ2 might be used in other continents than Africa where exposure to *P. falciparum *is low.

In 2008, a pilot programme for the evaluation of blister-packed treatment in distinct ethnic groups was initiated. An initial cross-sectional study was carried out in two indigenous population groups in order to gain knowledge about the micro-epidemiology of malaria in the project area. A second cross-sectional study is planned to close the project. As serological markers are useful indicators to measure transmission variations especially in low endemicity areas [12], immune responses to MSP3 and GLURP antigens were investigated as indicators of naturally acquired immunity to *P. falciparum *antigens.

## Methods

### Study population

The study took place in June 2009 in the municipality of Atures, Amazonas, Venezuela. Three indigenous communities were visited. They were inhabited by two ethnic indigenous population groups, the Guahibo (also: Guajibo, Wahibo, Hiwi, Jivi) and the Piaroa: 1) Platanillal (475 inhabitants, Guahibo); 2) Cerro de Oro (60 inhabitants, Guahibo); 3) Paria Grande (463 inhabitants, Piaroa). The distance between the communities is small (less than 20 km linear distance) and they are comparable with respect to ecological, geographical and malariological conditions such as distance to *Anopheles *breeding sites. The majority of residences in the communities are non-traditional governmental housing projects. The communities of Platanillal and Paria Grande have a health post, each with a malaria microscopist.

### Ethical clearance, informed consent, and treatment

Ethical clearance was obtained from the institutional ethical committee of the Amazon Center for the Investigation and Control of Tropical Diseases 'Simón Bolívar', Autonomous Service, Puerto Ayacucho, Amazon State, Venezuela (SACAICET). Residents were informed about the ideas and procedures of the study, when necessary with the help of translators. All residents were invited to take part. Those consenting orally were interviewed and examined. Individuals with a blood smear positive for malaria were treated according to the national guidelines (*P. falciparum: *artesunate, mefloquine, and primaquine. *P. vivax: *chloroquine, primaquine) [13].

### Data extraction of the regional malaria documentation system

Data from the regional malaria documentation system were extracted. Malaria cases detected in the health posts of Platanillal and Paria Grande before the study took place, from January 2003 to May 2009, were analysed.

### Interviews and basic physical examination

Every participant, or the legal guardian, was interviewed using semi-structured interviews. Translators assisted if it was necessary, and standardized questions were phrased with the support of anthropologists. The structured part of the interviews concerned malaria antecedents, possession and use of mosquito nets, general condition, unspecific symptoms during the last seven days, and pregnancy. Every participant was examined physically including a check for splenomegaly and body temperature.

### Smears and serum

Thick blood smears and thin films were taken from every participant, air-dried and stained for 45 min with 3% Giemsa using standard procedures1 [14]. Slides were read and re-read by trained professionals of the SACAICET and of the Regional Health Directory in Puerto Ayacucho. Ten ml of blood was taken from every participant into a vacutainer and centrifuged. Serum was stored at −20°C until further processing.

### Recombinant antigens

Recombinant proteins were produced in *Escherichia coli *as described elsewhere [15]. The 48 kDa merozoite surface protein 3 (MSP3) is divided into two major allele types by sequence polymorphism. The C-terminal region of the molecule is highly conserved [16]. The 220 kDa glutamate-rich protein (GLURP) contains one non-repeat and two repeat regions. Both antigens are involved in antibody dependent cellular inhibition (ADCI) and are used as sub-units of the GMZ2 fusion protein [17]. For all experiments the conserved fragments MSP3_212-318 _and GLURP_27-500 _were used.

### Antibody measurements

Serum antibodies to MSP3 and GLURP were measured applying a modified version of a quantitative ELISA [18]. In brief, microtitre plates were coated with antigens in a dilution of 0.5 mg/ml in PBS overnight at 4°C. After a first washing cycle (3 times) with PBS-T plus 0,5 mol/litre NaCl wells were blocked for one hour with a dilution of 30 g of defatted milk powder per litre PBS-T. As standard, after another washing cycle, duplicate rows were coated with samples, controls and a standard sample (with quantified high antibody concentration) in blocking buffer for two more hours. The standard sample was added in an X-fold serial dilution from 1/20 to 1/327,680. Samples and controls were added in duplicates in dilutions of 1/50 and 1/200. Samples with optical densities higher than the positive control were repeated at 1/800. After washing again, plates were incubated with peroxidase conjugated goat anti-human IgG (Caltag, 1/5,000) for one hour, washed, treated with TMB-One for 15 min in gloomieness until the reaction was stopped with sulphuric acid. Then absorbance was read at 450 nm. The standard sample was used to calculate IgG concentration of the other samples in mg/dl. Pooled serum from 20 individuals, never having lived in malaria-endemic areas, served as negative control. The arithmetic mean of negative controls was 0.0746 mg/dl (SD 0.0396 mg/dl) and 0.092 mg/dl (SD 0.0441 mg/dl) for MSP3 and GLURP, respectively.

### Data capture and analysis

All data were subjected to plausibility, double-entered and checked for transcription errors in Epidata v3.0^®^. Optical densities (ODs) were averaged and normalized. The cut-off value for the difference between duplicates was set at 25%. If the difference was higher, the measurement was repeated. All results were converted to values in mg/dl using the positive standard serum. Positivity and high positivity of samples was defined as any OD higher than the mean of negative controls plus three and five standard deviations (SD), respectively. Statistical analysis was performed using SPSS 19^® ^software. T-Tests were run to screen for significant differences between the variables subgroups concerning antibody titres, Chi-Square-Tests to learn about disparities in respect to antibody positivity. Age in years, sex, self-reported incidence of malaria episodes during the last 12 months and lifetime, possession and frequent utilization of mosquito nets and ethnic affiliation were included in a binary logistic model to predict anti-MSP3 and anti-GLURP antibody positivity. This quite conservative model has the advantage to show an Odds ratio (OR) for each expression of a variable. Furthermore, a linear regression model was performed to expose the influence of the aforementioned independent variables on the direct IgG-titre against the GMZ2-subunits.To do so, antibody levels had to be transformed to their decadic logarithm to gain Gaussian distribution. The resulting model was then de-logarithmized to show comparable and comprehensible results.

## Results

### Study population

Four hundred and seventy-five persons participated in the study. Demographic data of participants and questionnaire results are summarized in Table 1.

**Table 1:** Demographic data of participants and questionnaire results

	Guahibo (n = 180)	Piaroa (n = 295)
Age (years, SD)	20,8 (SD 19,1)	22,4 (SD 16,8)
Sex (female)	108 (60%)	147 (49,8%)
Possession of mosquito net	143 (79,4%)	285 (96,6%)
Constant use of mosquito net	125 (69,4%)	263 (89,2%)
Malaria episode in last 12 months	32 (17,8%)	36 (12,2%)
Malaria episode ever	112 (62,2%)	170 (57,6%)

### Malaria infections in the two indigenous communities from 2003 to 2008

#### Platanillal

Within the six years preceding the cross-sectional study (2003 - 2008), the health post of Platanillal reported 573 cases of malaria in Guahibo patients (2003: 134 cases; 2004: 62 cases; 2005: 90 cases; 2006: 141 cases; 2007: 95 cases; 2008: 51 cases).

#### Paria grande

The health post of Paria Grande reported 270 cases of malaria in Piaroa patients within the six years preceding the cross-sectional study (2003: 11 cases; 2004: 65 cases; 2005: 29 cases; 2006: 70 cases; 2007: 74 cases; 2008: 21 cases). Of these, 45/573 cases (7.8%) and 36/270 cases (13.3%) were caused by *P. falciparum *in Platanillal and Paria Grande, respectively. All other infections were caused by *P. vivax. P. malariae *was not detected.

### Malaria infections in the two indigenous communities from january 2009 to may 2009

#### Platanillal

Within the five months preceding the cross-sectional study (January 2009-May 2009), 154 slides were taken in the health post of Platanillal; 43/154 slides (27.9%) were positive for *P. vivax*, and 5/154 slides (3.2%) were positive for *P. falciparum*.

#### Paria grande

Within the five months preceding the cross-sectional study, 148 slides were taken in the health post of Paria Grande; 15/148 slides (10.1%) were positive for *P. vivax*, and none (0%) was positive for *P. falciparum*.

#### Clinical results

Participants (n = 475) complained about the following symptoms within the preceding seven days: headache (71; 14,9%), body ache (45; 9,5%), flu-like symptoms (101; 21,3%), cough (34; 7,2%), chills (5; 1,1%), fever (28; 5,9%), diarrhoea (46; 9,7%), abdominal pain (30, 6,3%), nausea (34, 7,2%), fatigue (14, 2,9%). The mean temperature of all participants was 37,02°C (Min. 35,8°C, Max. 38,9°C, SD. 0,4803). 9/475 participants (1.9%) had a body temperature over 38°C. None of the participants had palpable splenomegaly and none of the 475 participants was found positive for *P. falciparum *at the day of the cross-sectional study. Two participants (2/475; 0.4%) were found positive for *P. vivax *(both <100 parasites/μl). Both patients were from the Guahibo ethnic group and reported malaria antecedents. One patient was a 12-year-old anaemic boy (Hb 12.5 mg/dl) in reduced general condition and with normal body temperature (36.5°C) reporting chills during the last seven days. The other patient was a 26-year-old anaemic pregnant woman (Hb 9.7 mg/dl) with normal body temperature (37.1°C) complaining about a reduced general state of health.

#### Antibodies to MSP3 and GLURP

The cut-off value for a positive sample was set at 0.193 mg/dl and at 0.224 mg/dl for MSP3 and GLURP, respectively. 111/475 participants (23.4%) were positive for at least one of the antibodies. 53/475 participants (11.2%) were positive for MSP3, and 93/475 participants (19.6%) were positive for GLURP. 35/475 participants (7.4%) had positive antibody levels against both antigens. The average value (geometric mean) of positive samples was 0.36 mg/dl (95% CI 0.32 mg/dl - 0.41 mg/dl) and 0.47 mg/dl (95% CI 0.41 mg/dl - 0.55 mg/dl) for MSP3 and GLURP, respectively. The cut-off value for a high-positive sample was set at 0.27 mg/dl and at 0.31 mg/dl for MSP3 and GLURP, respectively. 78/475 participants (16.4%) were high-positive for at least one of the antibodies. 36/475 participants (7.6%) were high-positive for MSP3, and 61/475 participants (12.8%) were high-positive for GLURP. 19/475 participants (4%) had high-positive antibody levels for both antigens. The highest titres measured were 2.8 mg/dl and 3.36 mg/dl for MSP3 and GLURP, respectively.

#### Stratification for age and sex

Antibody titres were significantly increasing with age (Spearman Rank Test, R_SpGLURP _= 0.130, p = 0.005. R_SpMSP3 _= 0.259, p < 0.001). When the study population was grouped by age - each group with a span of 10 years - a significantly higher number of positives and high-positives was seen in the older age groups (ANOVA: positives: p_MSP3 _< 0.001, p_GLURP _= 0.001, high-positives: p_MSP3 _< 0.001, p_GLURP _= 0.028). No significant differences in antibody titres or positivity rates were found regarding the sex of the persons (*T*-test: p_GLURP _= 0.101, p_MSP3 _= 0.169, Chi-square: p_GLURP _= 0.101, p_MSP3 _= 0.184).

#### Stratification for possession and use of mosquito nets

Comparing possessors (428/475, 90.1%) and non-possessors (47/475, 9.9%) of mosquito nets, holders were found to have lower levels of GLURP (*T*-test, p_GLURP _< 0.001), but there was no significant difference respective to MSP3 (*T*-test, p_MSP3 _= 0.489). Participants who reported daily use of mosquito nets during the previous 30 nights were defined frequent users. 392/475 participants (82.5%) were found to be frequent users. No significant difference in antibody titres nor positivity rates were found between frequent users and the rest of the study population (*T*-test, p_GLURP _= 0.125, p_MSP3 _= 0.991; Chi-square-test, p_GLURP _= 0.182, p_MSP3 _= 0.084).

#### Stratification for self-reported malaria episodes

282/475 participants (59.4%) stated they had at least one malaria episode in their life, and 85/475 (17.9%) reported a malaria episode during the previous 12 months. Those who reported to have suffered malaria at least once in their life showed significantly higher IgG-levels than the group reporting no previous malaria episode. This difference was significant for MSP3 (*T*-test, p_MSP3 _= 0.032), but not for GLURP (*T*-test, p_GLURP _= 0.062). Those who stated a malaria episode within the previous 12 months had slightly more positive responses against both antigens. This difference was significant for GLURP and for MSP3 (Chi-square, p_GLURP _= 0.002; p_MSP3 _< 0.001).

#### Stratification for ethnic origin

Participants from the ethnic Guahibo group displayed significantly higher antibody titres and positivity rates for both antigens when compared to the ethnic Piaroa group (Table 2).

**Table 2:** Antibody titres and positivity rates in Guahibo and Piaroa participants

	Guahibo (n = 180)		Piaroa (n = 295)	
	
	mg/dl (95% CI)	Pos (%)	mg/dl (95%)	Pos (%)
MSP3	0.07^1 ^(0.06-0,09)	44^2 ^(24,4%)	0.05^1 ^(0.05-0.08)	9^2 ^(3.1%)
GLURP	0.10^3 ^(0.09-0.13)	54^4 ^(30%)	0.07^3 ^(0.07-0.08)	39^4 ^(13.2%)

^1 ^*T*-test, p = 0.001; ^2 ^Chi-square, p < 0.001; ^3 ^*T*^-test^, p = 0.002; ^4 ^Chi-square, p_GLURP _< 0.001

#### Binary logistic and linear regression model

The above-mentioned parameters were included in a binary logistic and a linear regression model to predict positivity and titres of antibodies against MSP3 and GLURP. In the binary logistic model, the parameters age, possession of mosquito nets, and ethnic origin had a significant outcome: For every year of age, risk of positivity raised with an OR of 1.03 for both MSP3 (CI: 1.018; 1.051, p < = 0.001) and GLURP (CI: 1.020; 1.049, p < = 0.001). Non-possession of a mosquito net increased the chance to have GLURP by 4.05-fold (CI: 1.258; 13.014, p < = 0.001). Guahibo individuals had a 8.04-fold (CI: 4.061; 15.898, p < = 0.001) chance for MSP3 antibody positivity compared to Piaroa individuals. With respect to GLURP, the chance for positivity was 2.81-fold higher for Guahibo in comparison to Piaroa individuals (CI: 1.677; 4.721, p < = 0.001). Results of the linear regression model are shown in Table 3.

**Table 3:** Linear regression model

	MSP3				GLURP			
	
	β	95% low	CI for β high	p	β	95% low	CI for β high	p
Age in years	1,02	1,01	1,02	<0.001	1,01	1,00	1,01	0.019
Sex	1,07	0,92	1,26	0.373	1,10	0,90	1,36	0.354
Possession mosquito net	1,12	0,77	1,63	0.556	2,52	1,54	4,13	<0.001
Constant use of ITN^1^	1,18	0,89	1,56	0.256	1,30	0,90	1,88	0.168
Malaria episode (ever)	1,10	0,92	1,32	0.309	0,99	0,78	1,26	0.947
Malaria episode (12 m^2^)	1,37	1,08	1,73	0.009	1,34	0,98	1,82	0.066
Ethnic group	1,31	1,10	1,54	0.002	1,26	1,01	1,57	0.041

Explanation: As antibody titres are not expected to be Gaussian distributed the models calculated with the decadic logarithms of the measured IgG levels. Results were then delogarithmized. Age in years showed significant influence for each year of age. Females' trend to have higher antibody levels resulted not statistically significant. The possession of a mosquito net caused no significant coefficient for MSP3, but for GLURP, whereas every night usage of a mosquito-net during the last 30 days entailed no significant influence antibodies. The coefficients for self-reported malaria episodes during the whole lifetime were not significant, unlike those for the incidence in the previous 12 months (MSP3). Ethnic background affected the linear equation with significant coefficients for both, MSP3 and GLURP

^1 ^Insecticide treated net

^2 ^Within the last 12 months

## Discussion

Results show that the two GMZ2 antigens are recognized by antibodies acquired through natural infections in the Venezuelan Amazon. Overall, the results correspond - except high responders - to rather low responses which are roughly two-fold higher than the cut-off value for positives. As antibody subclasses are of great importance [19] further studies on IG-subclasses have to show whether functional antibodies are acquired through natural exposure. The question if a GMZ2 vaccine would be effective in this area remains open, because antibodies stimulated by vaccines do not automatically protect as do naturally acquired antibodies [20]. In respecto to vaccine efficacy, only clinical trials with disease as endpoint can prove the impact of a vaccine.

The two main findings of this study were 1) the high prevalence of antibodies against conserved parts of MSP3 and GLURP antigens in the two population groups with low incidence of *P. falciparum *malaria, and 2) the significantly higher prevalence of MSP3 and GLURP antibodies in the Guahibo group when compared to the Piaroa group.

The total population of the Piaroa is about 12,000 persons. Anthropologists describe them as peaceful and egalitarian. Traditionally, the Piaroa's agroforestal subsistence strategy implicated small, dispersed and mobile ways of settlement in groups of about 50 individuals. Nowadays they are living in bigger communities mostly built by the Venezuelan government in the periphery of their ancestral territories, which they still use as cropland and hunting grounds [21].

The Guahibo consist of about 30,000 persons settling mainly around Puerto Ayacucho and neighbouring Colombian areas. As a patriarchal society, great importance is placed on the nuclear family. Traditionally, they inhabited vast flatlands in Colombia and Venezuela as nomadic foragers. Nowadays, they inhabit communities constructed by the Venezuelan government. They are mainly assimilated according to language and clothing style, but still are threatened by pauperisation [22].

The serological results of this study confirm previous investigations hinting to an elevated risk for malaria in the Guahibo population [2,23]. Living conditions, socioeconomic parameters, adherence to medicaments, confidence in the health system, and cultural habits may explain the different transmission levels in the two ethnic groups under study. Different immune responses with different protection patterns in genetically different populations are also a possible factor [24,25]. Further research involving multidisplinary expertise as well as members of the communities should focus on this "hot spot" of malaria transmission in the Venezuelan Amazon.

The positive correlation of antibody titeres and prevalences with age has been known for a long time [26]. When people live in an area endemic for malaria the chances of contact with the antigen raise with the years, and so do antibody titres and prevalences. Similarly, the chances of contact decrease with the use of mosquito nets. Such, as shown in other studies [27], a difference in GLURP antibody titres and prevalences could be observed between mosquito net users and non-users.

This cross-sectional study evaluated immune responses against two blood stage antigens considered as promising vaccine candidates on the basis of studies carried out in completely different settings. Samples were taken in the period of low transmission when the direct contact with *P. falciparum *parasites is expected to be minimal. Indeed, only two persons in nearly half a thousand participants were found harbouring parasites (2/475; 0.4%). Both patients were positive for *P. vivax*, none was positive for *P. falciparum*. Nevertheless, nearly a quarter of participants had antibodies to at least one of both antigens. As adding up immune responses to individual antigens might not be an appropriate procedure for results, which show - not surprisingly for outbred human populations - an extensive variation between individuals, it is important to note that 7.6% and 12,8% of participants had high antibody titres against MSP3 and GLURP, respectively. Possibly only high-positive antibody responses are relevant and/or specific results in this endemic area. Moreover, it should be taken into account that higher antibody responses might persist longer than lower ones.

Using the records of the health information system of Amazonas the level of malaria transmission in the communities during the years and months before the study was approximated. As the health posts of Platanillal and Paria Grande were comparable in the volume of slides taken per year (e.g. 2009: Platanillal, 280 slides; Paria Grande, 269 slides) and data of ethnic origin, origin of infection, and population census were available, the prevalence in both communities could be estimated for malaria in general, and for *P. falciparum *in particular. The average pan-malarial incidence was higher in the Guahibo population of Platanillal (*ca *18%) than in the Piaroa population of Paria Grande (*ca *8%). However, this was due to *P. vivax *infections, whereas the transmission of *P. falciparum *was surprisingly low in both population groups. Within a time period of 6.5 years, only 50 and 36 cases of *P. falciparum *were detected in Guahibo and Piaroa people of the health posts of Platanillal and Paria Grande, respectively, and the average incidence of *P. falciparum *was estimated to be less than two percent in both population groups, without a significant difference.

MSP3 and GLURP antibodies last six-12 months if no boost infection takes place [8]. In order to explain the phenomenon that antibodies were detected in more individuals than expected, submicroscopical infections must be considered as an influencing variable. The use of other antigens (for example, pre-erythrocytic antigens) might have led to other results, yet it can be assumed that conclusions would have differed slightly only. This is in line with previous investigations demonstrating asymptomatic carriers in this area [28]; interestingly, more than 20% of those who denied they ever suffered from any type of malaria had positive IgG titres. Another - yet to be proven - hypothesis would be the polyclonal activation of effector T killer cells and memory B-cells against *P. falciparum *antigens by *P. vivax *[29].

Controlled malaria is the result of an interplay of host defense systems, public health measures, ambiental conditions, behavioural features, and epidemiology and infection biology of the parasite. Interventions should be weighed carefully in order not to destabilize an established equilibrium. The epidemiology and immunology of controlled malaria might be subject of growing interest for malariologists and decision makers, as lessons learnt in low endemic areas could be instructive after eradication and control efforts have been successful in high endemic areas.

## Competing interests

The authors declare that they have no competing interests.

## Authors' contributions

AB coordinated and conducted field and laboratory work, analysed and interpreted the data, and drafted and revised the manuscript. MM conceived the study, supervized field and laboratory work and interpreted the data. SVM conceived the study, facilitated the overall collaboration and revised the manuscript. MLU and TH coordinated and conducted field and laboratory work. RD collaborated as anthropologist. ME and BGM supervized the laboratory work, gave statistical input, interpreted the data and revised the manuscript. MT and LA were responsible for the conception of the work. WGM conceived the study, interpreted the data, and drafted and revised the manuscript. All authors revised and approved the final manuscript. WGM and MM are the guarantors of the paper.
